# Endoscopic injection of human fibrin sealant in treatment of intrathoracic anastomotic leakage after esophageal cancer surgery

**DOI:** 10.1186/s13019-020-01127-w

**Published:** 2020-05-14

**Authors:** Xueyu Chen, Xiaoqin Yuan, Zhongyuan Chen, Lianggang Zhu

**Affiliations:** 1 Department of Thoracic Surgery, Ruijin Hospital North, Shanghai Jiaotong University School of Medicine, Shanghai, 201801 China; 2 Department of Gastroenterology, Ruijin Hospital North, Shanghai Jiaotong University School of Medicine, Shanghai, 201801 China

**Keywords:** Esophageal cancer, Anastomotic leakage, Fibrin sealant

## Abstract

**Objective:**

To investigate the application of endoscopic injection of human fibrin sealant in treatment of patients with intrathoracic anastomotic leakage after esophagectomy.

**Methods:**

A total of 179 patients who underwent intrathoracic anastomosis after esophageal cancer surgery in our department From December 2012 to May 2015 were retrospectively analyzed. The clinical data and treatment of 7 patients with postoperative intrathoracic anastomotic leakage were analyzed and discussed. On Day 28 after operation, the 7 patients were given endoscopic injection of human fibrin sealant to seal the anastomotic leakage, and the changes in drainage volume, body temperature, CRP, white blood cell count and other indicators were compared before and after endoscopic intervention.

**Results:**

After endoscopic injection of human fibrin sealant in all 7 patients with intrathoracic anastomotic leakage, the volume of para-anastomotic drainage, CRP, and WBC count were improved compared with those before treatment. Relevant data were analyzed, and the differences were statistically significant (*P* = 0.019, *P* = 0.001, *P* = 0.014, respectively). No statistically significant difference was observed in the body temperature before and after treatment (*P* = 0.217).

**Conclusion:**

For patients with intrathoracic anastomotic leakage after esophageal cancer surgery, endoscopic injection of human fibrin sealant to seal the anastomotic leakage has positive therapeutic effects of reducing exudation around the anastomotic leakage, reducing systemic inflammatory response, and improving clinical symptoms including dysphagia, weight loss without trying, chest pain, pressure or burning, worsening indigestion or heartburn and coughing or hoarseness.

## Background

At present, the incidence rate of esophageal malignant tumor is about 13/100,000 in China, ranking first in the world [1, 2]. Surgical resection of the lesion along with reconstruction of the digestive tract is the main treatment to prolong the survival time of patients and improve their quality of life. Postoperative anastomotic leakage has been one of the main causes of poor prognosis in patients, which plagues thoracic surgeons [3, 4]. With the continuous improvement of surgical techniques and anastomosis methods, the incidence of anastomotic leakage after esophageal cancer surgery is generally 1.0–5.5% as reported in recent years in China [5]. If not treated effectively in time, the condition may further develop and worsen, inducing induced MODS, especially intrathoracic anastomotic leakage, with a higher mortality than other gastrointestinal anastomotic leaks, which is the main cause of perioperative mortality in esophageal cancer and the characteristics included that The time of anastomotic leakage such as early leakage, mid-term leakage and late leakage found was 4 to 45 days, with a median time of 10 days. The anastomotic leakage had a company with tracheoesophageal fistula and contralateral pleural fistula [6, 7]. A total of 179 patients underwent radical resection of intrathoracic anastomotic esophageal cancer in our department from December 2012 to May 2015, and 7 of them had postoperative intrathoracic anastomotic leakage. Based on routine fasting, anti-inflammatory treatment, and nutritional support treatment for these 7 patients, the anastomotic leakage was closed by endoscopic injection of fibrin sealant. The clinical data were retrospectively analyzed, and the results are reported as follows for the reference of clinical peers.

## Materials and methods

### General information

Among the patients with esophageal cancer admitted from December 2012 to May 2015, a total of 179 patients with surgical indications who underwent radical esophagectomy (two- or three-field lymph node dissection + intrathoracic anastomosis) after preoperative examination and evaluation. These patients had an oval silicone vacuum suction tube with a length of about 5 mm placed beside the anastomosis in addition to the conventional placement of a closed thoracic drainage tube. These cups were not round but oval. Silicone cups had a shore hardness between 54 and 80 HS. They could be used between − 60 °C and 250 °C and were particularly indicated for food and food packaging appliances. Seven patients had postoperative intrathoracic anastomotic leakage, with an incidence of 3.91%, among which were 5 males and 2 females, aged 49–72 years, with a mean of 59.6 ± 7.9 years. Pathologically, all the 7 patients developed squamous cell carcinoma, with 2 cases of pathological stage IIA, 3 cases of stage IIB, and 3 cases of stage IIIA.

### Clinical symptoms and diagnosis

About 1 week after operation, 7 patients had clinical manifestations of low-grade fever, chest tightness, chest pain, palpitation, and shortness of breath. Increased WCB and CRP indicators were observed in all patients. Daily drainage volume beside the anastomosis was significantly increased than before, and the drainage fluid was turbid with foul smell. Anastomotic leakage was confirmed in 7 patients after oral administration of 50 mL of normal saline containing methylene blue dye solution, and blue dye solution flowed out from the drainage tube adjacent to the anastomosis.

### Treatment

All the 7 patients were fasted and were given gastrointestinal decompression, intravenous and enteral nutrition support, and broad-spectrum antibiotics to prevent and control infection. Chest CT scan was used to check whether there was obvious pleural effusion and the unsmooth drainage. At the same time, the drainage fluid of the patient’s anastomotic drainage tube was sent for bacterial and fungal examination, and the antibiotics were adjusted according to the results of drug sensitivity test. In case of drug tolerance, the dose of enteral nutrition was increased as much as possible to provide adequate enteral nutrition support.

**Fig. 1:**
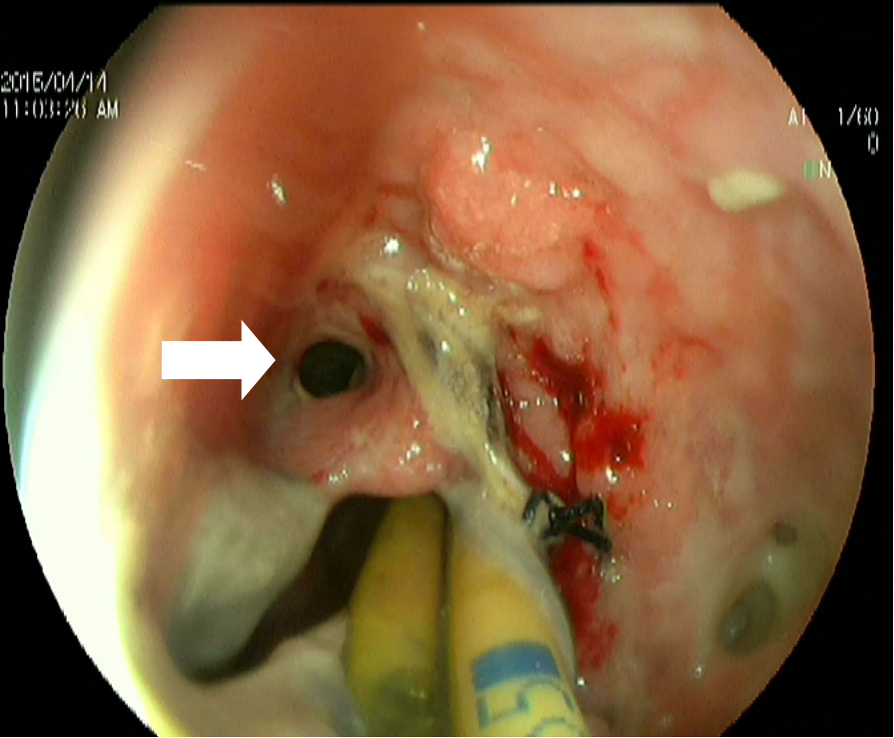
The arrow points to the anastomotic leak

**Fig. 2:**
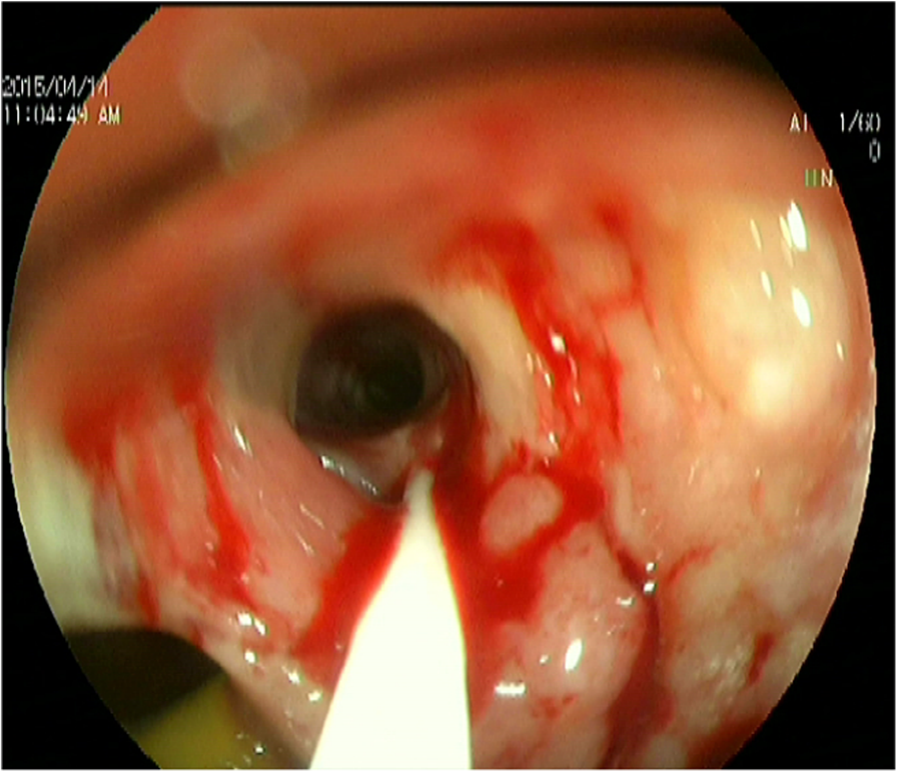
Fibrin sealant is injected into the leak

**Fig. 3:**
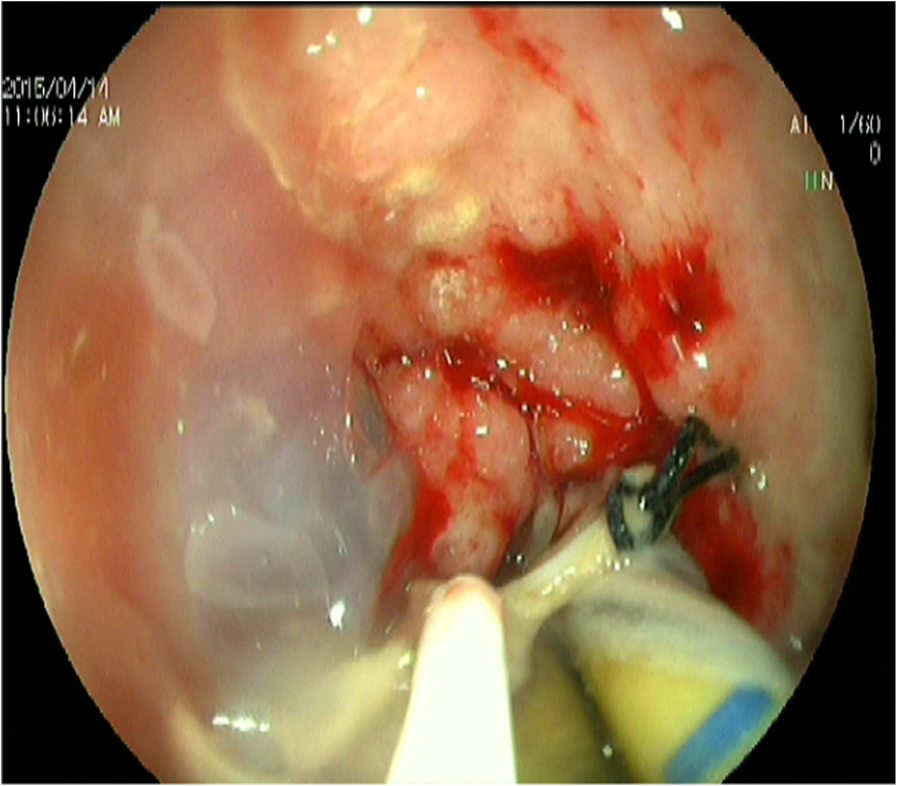
The anastomotic leak is essentially closed after injection

On Day 28 after operation, the above patients received gastroscopy, and 7 patients were found to have anastomotic leakage of tubular stomach and esophageal stump under gastroscope. The size was about 3–6 mm in diameter by visual inspection under microscope. After washing the white hair around the anastomotic leakage (Fig. 1), a tailored endoscopic injection extension tube was inserted into the patient’s stomach through the forceps channel of the gastroscope, which was then connected to the syringe containing the mixed lyophilized human fibrin sealant (FIBINGLURAAS, Shanghai RAAS Blood Products Co., Ltd.). The endoscopist extended the extension tube approximately 0.5 cm into the anastomotic leak, approximately at the tubular gastric serosal layer (Fig. 2), and slowly injected half of the syringe containing the lyophilized human fibrin sealant back into the extension tube to fill the serosal layer to the mucosal layer of the tubular stomach completely with the fibrin sealant, thereby closing or reducing the anastomotic leakage (Fig. 3). In general, fibrin sealant became the first modern era material approved as a hemostat in the United States in 1998. It is the only agent presently approved as a hemostat, sealant, and adhesive by the Food and Drug Administration (FDA). The product is now supplied as patches in addition to the original liquid formulations. Both laboratory and clinical uses of fibrin sealant continue to grow [8]. In this study, the fibrin sealant was applied 3 weeks after detection of the leak just due to the haematemesis before 3 weeks.

### Observation indicators

The mean body temperature (measured at 6 o’clock in the morning each day) and the mean anastomotic drainage volume of these 7 patients before and after endoscopic injection of fibrin sealant was collected. The WBC count and CR*P* value measured within 1 week before and after treatment were collected as the paired data before and after treatment.

### Statistical methods

All statistical analyses were perform using SPSS 15.0 software. The parameters of each sample were tested for normality, and the results showed that they conformed to a normal distribution and described as $\overline{x}\pm s$. The paired data of 7 patients before and after endoscopic treatment were collected, and the means of two samples in paired design were compared (paired t test). *P* < 0.05 was considered as statistically significant differences.

## Results

All 7 patients with postoperative intrathoracic anastomotic leakage received endoscopic injection of fibrin sealant, with an average treatment time of 23.8 ± 8.9 min. After treatment, the mean daily para-anastomotic drainage volume decreased in 7 patients, and the difference was statistically significant (*P* = 0.019). However, the changes in mean body temperature before and after treatment showed no significant difference (*P* = 0.217). After treatment, the WBC count was significantly lower than before treatment, and the difference was statistically significant (*P* = 0.001). The change in CRP also showed a similar change to WBC count, which was statistically different from the value before treatment (*P* = 0.014), as shown in Table 1. After receiving endoscopic treatment for 1 month, 6 patients underwent radiography with oral water-soluble contrast agent, and no contrast agent leakage was found, suggesting that the anastomotic leakage was basically healed. All patients were discharged within 45 days after food consumption without any abnormality. One patient with contrast agent leakage underwent another gastroscopy, and the anastomotic leakage was significantly smaller than before. The patient received additional fibrin sealant injection, and was recovered and discharged after 20 days.

**Table 1:** Paired comparison of indicators before and after treatment in 7 patients

	Before treatment	After treatment	*P* value
Mean drainage volume (mL/d)	82.1 ± 16.7	40.7 ± 7.6	0.019*
Mean body temperature (°C)	37.4 ± 0.1	37.2 ± 0.3	0.217
White blood cell count (×10^9/L)	11.0 ± 1.6	9.0 ± 1.3*	0.001*
Blood CRP (mg/L)	33.7 ± 4.8	24.3 ± 4.6*	0.014*

Compared with the value before treatment, *P* < 0.05 was considered as significant difference

## Discussion

Intrathoracic anastomotic leakage has been a serious complication after radical resection of esophageal cancer [9, 10]. Improper treatment will induce other serious postoperative complications including thoracic infection, empyema, MODS, and septic shock, and can even be life-threatening in severe cases [11, 12]. There are many causes of postoperative anastomotic leakage, including the anatomical characteristics of the esophagus itself, such as no serosal covering of the esophagus, longitudinal muscle fibers, and easy tearing at the same direction of suturing and pulling [13], anastomotic blood supply, and anastomotic tension. The surgical patients were additionally indwelled with negative pressure drainage tube adjacent to the anastomosis, so the drainage volume and color of drainage fluid of this drainage tube could be observed every day to identify abnormal conditions in a prompt manner, and suggest the occurrence of anastomotic leakage [14]. Besides, this drainage tube allows the removal of the patient’s thoracic closed drainage tube in the early postoperative period to facilitate the patient’s early ambulation to promote recovery. Closed thoracic drainage tube is placed at a low position after traditional esophageal cancer surgery [15]. When more digestive juice leaks out into the thoracic cavity, it will flow to the thoracic drainage tube. Generally, the systemic inflammatory response of the patient is more obvious or even serious currently. However, the additional silicone tube was located at the esophageal stump and near the tubular gastric anastomosis after operation. Therefore, in the early stage of anastomotic leakage, when a very small amount of digestive juice leaks out of the leakage, it can flow out from the drainage tube next to the anastomosis, and the drainage effect is more ideal than that of simple closed thoracic drainage [16]. Moreover, the anastomotic leakage can also be found more easily by clinicians in the early stage, which avoids the anastomotic leakage from further expanding with the continuous leakage of digestive juice in the tubular stomach, and increases the difficulty of endoscopic treatment in the later stage [17]. In this study, 7 patients were found to have anastomotic leakage in the early stage due to indwelling single-lumen drainage tube beside the anastomosis during the operation, and none of them had severe infection or poisoning symptoms. No local package or poor drainage was found by chest CT, and the diameter of anastomotic leakage was generally smaller in the subsequent endoscopic examination, increasing the success rate and effectiveness of endoscopic treatment.

In the past, when intrathoracic anastomotic leakage was found after radical resection of esophageal cancer, active reoperation was performed to repair the leakage. However, it still had a high recurrence rate and mortality after operation, which has been rarely used as the first choice of treatment. In recent years, increasing scholars have reported the experience of using esophageal stent to treat anastomotic leakage when intrathoracic anastomotic leakage occurs after esophageal cancer surgery [18–20], and a certain therapeutic effect has been achieved. However, some literatures have also indicated that the implanted esophageal stent may puncture the esophageal mucosa to cause bleeding, and result in displacement, failure to seal the fistula, stenosis caused by granulation, and other related problems [20, 21]. Additionally, it is easy to cause patient discomfort.

In this study, fibrin sealant was extracted from human blood, which avoided the immune rejection and hypersensitivity caused by heterologous protein since human tissue has a strong adaptability. Endoscopic injection can seal the anastomotic leakage, block the connection between tubular stomach and thoracic cavity through anastomotic leakage, and reduce the irritation of inflammatory factors and digestive juice to local mucosa. Meanwhile, the semi-solid gel formed has certain malleability and elasticity, which will not affect the normal peristalsis of digestive tract, nor have the foreign body sensation of esophageal stent and the risk of bleeding caused by damage to esophageal mucosa. As early as 2000, Pross M, et al. [22] performed occlusion in 7 patients with anastomotic leakage and achieved success, suggesting that the application of biological fibrin glue for medical use to block the leakage under endoscope is a relatively safe and less invasive method. Jia Tao et al. [23] used biological fibrin glue combined with gelfoam to treat anastomotic leakage after esophageal cancer surgery, which also achieved good therapeutic effect.

In this study, 7 patients with anastomotic leakage received gastroscopy on about Day 28 after operation. The time period was selected based on our experience. At this time, the anastomotic mucosa other than the leakage had basically healed well, with certain tensile strength, and could tolerate gastroscopy without causing iatrogenic injury. At the same time, the balloon was inflated under endoscope to meet the exposure of tubular gastric lumen, to avoid continuous large amount of inflation to generate tension around the anastomotic leakage and expand the scope of leakage. In this study, 7 patients had a total volume of 8 mL after mixing a set of fibrin sealant since the anastomotic leakage was not large during endoscopic treatment, which was enough for sealing the leakage in one treatment. None of the 7 patients had obvious discomfort after treatment, and the volume of single-lumen drainage beside the anastomosis decreased significantly from the next day after treatment compared with that before treatment. It was the injection of fibrin sealant under endoscope that sealed up the anastomotic leakage or reduced the diameter of anastomotic leakage to a certain extent, and reduced the amount of digestive juice flowing out through the leakage in tubular stomach. Therefore, the inflammatory irritation and local exudation of digestive juice to thoracic cavity were correspondingly reduced, and the systemic inflammatory response of the patient was also alleviated accordingly. The mucosal tissue and epithelial cells around the anastomotic leakage also got a clean and pollution-free environment due to the blocking of fibrin sealant, which promoted the repair of mucosa and the growth of epithelial cells. Therefore, the white blood cell count, CRP, and other indicators detected began to gradually decrease after treatment, and there were significant differences compared with those before treatment. In this study, there was no significant difference in the change of body temperature before and after treatment in 7 patients. The reasons were analyzed and may due to that all patients had received sufficient and effective chest drainage in the early stage of anastomotic leakage through the drainage tube around the anastomosis before treatment, and adjusted the antibiotic drugs accordingly in time based on the results of bacterial culture, so the perianastomotic and systemic inflammation of the patients had been controlled to some extent. In the meantime, the temperature measurement results of the patients were also susceptible to interference from the surrounding environmental factors and the stress response of the body after endoscopic treatment. Therefore, in this study, although the temperature measured in the 7 patients after endoscopic treatment was slightly lower than that before treatment, no significant difference in temperature change was obtained.

In 6 of the 7 patients, the anastomotic leakage basically healed within 45 days after treatment, and the daily anastomotic drainage volume was gradually reduced to 0–5 mL. After oral administration of water-soluble contrast agent, there was no obvious leakage of contrast agent. The patients were recovered and discharged after they gradually opened the diet until no obvious discomfort after eating semiliquid was found. One patient had a large anastomotic leak, approximately 6 mm in diameter. After sealing with fibrin sealant injected into the anastomotic leak, 30–47 mL of variable drainage fluid was still withdrawn from the drainage tube around the anastomosis daily. Nearly 2 months after endoscopic treatment, leakage of contrast agent was still observed after oral administration of water-soluble contrast agent, so another gastroscopy was arranged and a significant decrease in leakage of about 2 mm was observed. The patient was discharged from the hospital within 1 month after additional endoscopic injection. The anastomotic leakage was completely healed and the fibrin sealant was completely absorbed in the above patients during gastroscopy 30 days after discharge.

## Conclusions

We believe that in the case of postoperative anastomotic leakage in patients with esophageal cancer, in addition to conventional symptomatic treatment such as unobstructed drainage, reasonable use of antibiotics, and enhanced nutritional support, endoscopic examination and fibrin sealant injection and occlusion of anastomotic leakage can reduce the leakage of tubular gastric digestive juice through the anastomotic leakage, reduce the systemic inflammatory response of tissues and mucosa around the anastomotic leakage and patients, and have a positive therapeutic effect on the healing of intrathoracic anastomotic leakage after esophageal cancer surgery.

## Data Availability

All data generated or analyzed during this study are included in this published article.

## References

[1] Chen W, Zheng R, Zhang S, Zhao P, Zeng H, Zou X (2014). Report of cancer incidence and mortality in China, 2010. Ann Transl Med.

[2] Feng RM, Zong YN, Cao SM, Xu RH (2019). Current cancer situation in China: good or bad news from the 2018 global Cancer statistics?. Cancer Commun (Lond).

[3] Li X, Yan S, Ma Y, Li S, Wang Y, Wang X, et al. Impact of early oral Feeding on anastomotic leakage rate After esophagectomy: a systematic review and meta-analysis. World J Surg. 2020. Epub 2020/04/01. 10.1007/s00268-020-05489-z 32227277

[4] Mori M, Shuto K, Hirano A, Kosugi C, Narushima K, Hosokawa I, et al. A novel parameter identified using indocyanine green fluorescence angiography may contribute to predicting anastomotic leakage in gastric cancer surgery. World J Surg. 2020. Epub 2020/04/01. 10.1007/s00268-020-05488-0 32227275

[5] Smirnov AA, Vasilevskiy DI, Lapshin AS, Dvoretskiy SY, Filippov DI, Tsitskarava AZ, Bagnenko SF (2016). ANTIREFLUX RESECTION OF MUCOUS MEMBRANE OF ESOPHAGOGASTRIC ANASTOMOSIS IN TREATMENT OF BARRETT'S ESOPHAGUS: INITIAL EXPERIENCE. Vestn Khir Im I I Grek.

[6] Urschel JD (1995). Esophagogastrostomy anastomotic leaks complicating esophagectomy: a review. Am J Surg.

[7] Alanezi K, Urschel JD (2004). Mortality secondary to esophageal anastomotic leak. Ann Thorac Cardiovasc Surg.

[8] Spotnitz WD (2014). Fibrin sealant: the only approved hemostat, sealant, and adhesive-a laboratory and clinical perspective. ISRN Surg.

[9] Ahmed M, Habis S, Mahmoud A, Chin M, Saeed R (2019). Anastomotic leak after Esophagectomy for esophageal Cancer treated with a stent: a case report. Cureus.

[10] Verstegen MHP, Bouwense SAW, van Workum F, Ten Broek R, Siersema PD, Rovers M, Rosman C (2019). Management of intrathoracic and cervical anastomotic leakage after esophagectomy for esophageal cancer: a systematic review. World J Emerg Surg.

[11] Kang N, Zhang R, Ge W, Si P, Jiang M, Huang Y, Fang Y, Yao L, Wu K (2018). Major complications of minimally invasive Ivor Lewis oesophagectomy using the purse string-stapled anastomotic technique in 215 patients with oesophageal carcinoma. Interact Cardiovasc Thorac Surg.

[12] Kimura M (2016). Bypass operation for Unresectable esophageal Cancer: postoperative complications after thoracotomy versus no thoracotomy. Indian J Surg.

[13] Gooszen JAH, Goense L, Gisbertz SS, Ruurda JP, van Hillegersberg R, van Berge Henegouwen MI (2018). Intrathoracic versus cervical anastomosis and predictors of anastomotic leakage after oesophagectomy for cancer. Br J Surg.

[14] Umezawa H, Matsutani T, Yokoshima K, Nakamizo M, Ogawa R (2018). A novel tube-drainage technique of negative pressure wound therapy for fistulae after reconstructive surgery. Plast Reconstr Surg Glob Open.

[15] Loske G, Schorsch T, Muller CT (2017). Prevention of reflux after esophagectomy with endoscopic negative pressure therapy using a new double-lumen open-pore film drainage with an intestinal feeding tube. Endoscopy.

[16] Lin YH, Ou CY, Lee WT, Lee Y, Chang T, Yen YT (2019). Treatment outcomes for one-stage concurrent surgical resection and reconstruction of synchronous esophageal and head and neck squamous cell carcinoma. Eur Arch Otorhinolaryngol.

[17] Klink CD, Binnebosel M, Otto J, Boehm G, von Trotha KT, Hilgers RD, Conze J, Neumann UP, Jansen M (2012). Intrathoracic versus cervical anastomosis after resection of esophageal cancer: a matched pair analysis of 72 patients in a single center study. World J Surg Oncol.

[18] Persson S, Rouvelas I, Kumagai K, Song H, Lindblad M, Lundell L, Nilsson M, Tsai JA (2016). Treatment of esophageal anastomotic leakage with self-expanding metal stents: analysis of risk factors for treatment failure. Endosc Int Open.

[19] Eroglu A, Can Kurkcuogu I, Karaoganogu N, Tekinbas C, Yimaz O, Basog M (2004). Esophageal perforation: the importance of early diagnosis and primary repair. Dis Esophagus.

[20] Dai YY, Gretschel S, Dudeck O, Rau B, Schlag PM, Hunerbein M (2009). Treatment of oesophageal anastomotic leaks by temporary stenting with self-expanding plastic stents. Br J Surg.

[21] Schweigert M, Dubecz A, Stadlhuber RJ, Muschweck H, Stein HJ (2011). Risk of stent-related aortic erosion after endoscopic stent insertion for intrathoracic anastomotic leaks after esophagectomy. Ann Thorac Surg.

[22] Pross M, Manger T, Reinheckel T, Mirow L, Kunz D, Lippert H (2000). Endoscopic treatment of clinically symptomatic leaks of thoracic esophageal anastomoses. Gastrointest Endosc.

[23] Iizuka T, Kikuchi D, Yamada A, Hoteya S, Kajiyama Y, Kaise M (2015). Polyglycolic acid sheet application to prevent esophageal stricture after endoscopic submucosal dissection for esophageal squamous cell carcinoma. Endoscopy.

